# Advances in Alcoholism Treatment

**Published:** 2011

**Authors:** Robert B. Huebner, Lori Wolfgang Kantor

**Keywords:** Alcohol use disorders, alcohol dependence, alcoholism, treatment, treatment models, treatment research, treatment issues, cost-effectiveness, pharmacotherapy, medication therapy, behavior therapy, emerging technologies, co-treatment, 12-step-model, continuing care, health care delivery

## Abstract

Researchers are working on numerous and varied approaches to improving the accessibility, quality, effectiveness, and cost-effectiveness of treatment for alcohol use disorders (AUDs). This overview article summarizes the approaches reviewed in this issue, including potential future developments for alcoholism treatment, such as medications development, behavioral therapy, advances in technology that are being used to improve treatment, integrated care of patients with AUDs and co-occurring disorders, the role of 12-step programs in the broader realm of treatment, treating patients with recurring and chronic alcohol dependence, strategies to close the gap between treatment need and treatment utilization, and how changes in the health care system may affect the delivery of treatment. This research will not only reveal new medications and behavioral therapies but also will contribute to new ways of approaching current treatment problems.

Alcoholism treatment, as it exists today, rests on decades of research exploring the most effective ways to help people reduce their alcohol use or to stop drinking. That research has paved the way for the development and application of new methods and therapies and will continue to influence treatment practice in the future.

This article reviews the origins of alcoholism treatment and major studies of behavioral therapies and medications for treating alcohol dependence. It then provides a preview of the topics covered in this issue, including the potential future developments for alcoholism treatment such as medications development, behavioral therapy, advances in technology that are being used to improve treatment, integrated care of patients with alcohol use disorders (AUDs) and co-occurring disorders, the role of 12-step programs in the broader realm of treatment, treating patients with recurring and chronic alcohol dependence, strategies to close the gap between treatment need and treatment utilization, and how changes in the health care system may affect the delivery of treatment.

## Origins of Alcoholism Treatment

Alcoholics Anonymous (AA) was founded by Bill Wilson and Bob Smith in Akron, Ohio, in 1935. AA’s program of spiritual and character development, the 12 Steps, is based on the premise that turning one’s life and will over to a personally meaningful “higher power,” is the key to recovery. Another essential idea is that sobriety or recovery depends on the admission of powerlessness with respect to alcohol or other substances of abuse.

The Minnesota Model of addiction treatment was created in a State mental hospital in the 1950s. It was first practiced in a small nonprofit organization called the Hazelden Foundation In this approach, professional and trained nonprofessional (recovering) staff cooperated in applying the principles of AA. The model called for an individualized treatment plan with active family involvement in a 28-day inpatient setting and participation in AA both during and after treatment. Throughout the 1950s, Hazelden took the stance that (1) alcoholism is a disease and not a symptom of an underlying disorder and that it should be treated as a primary condition and (2) alcoholism affects people physically, mentally, and spiritually and that treatment for alcoholism should take all three aspects into account.

Around the same time that AA and Hazelden treatment methods were being refined and popularized, the study of alcohol abuse and alcoholism was expanding. Alcohol research, including the study of alcoholism treatment, found a home at the National Institutes of Health in 1970, when the National Institute on Alcohol Abuse and Alcoholism (NIAAA) was founded.

## Scope of the Problem

AUDs are prevalent in the United States and often go untreated. NIAAA’s National Epidemiologic Survey on Alcohol and Related Conditions (NESARC), a large general-population survey conducted in 2001–2002, estimated the prevalence of alcohol abuse and dependence at 4.65 percent and 3.81 percent, respectively (Grant et al. 2004).

Using NESARC results, Cohen and colleagues (2007) reported that only 14.6 percent of those with a lifetime history of alcohol abuse or dependence have received treatment. In another study that used NESARC results, Dawson and colleagues (2005) reported on people who experienced the onset of alcohol dependence at some point before the year prior to the survey. In this group, 25 percent still were alcohol dependent, 27.3 percent were in partial remission, 11.8 percent were in full remission but drinking at levels or patterns that put them at high risk for relapse, 17.7 percent were low-risk drinkers, and 18.2 percent were abstainers during the year prior to the survey.

Only 25.5 percent of these respondents reported ever receiving treatment. Among them, 3.1 percent participated in 12-step programs, 5.4 percent received formal treatment only, and the remaining 17 percent participated in both 12-step and formal treatment programs (Dawson et al. 2006).

Findings from this survey show that there is a wide range of recovery from alcohol dependence in the general population, from partial remission to full abstinence. The track of this disease is not clear cut—some people appear to recover from alcoholism without formal treatment. Others may cycle into and out of dependence throughout their lifetime despite repeated attempts to achieve sobriety (NIAAA 2006).

## Comparing Treatment Options: Project MATCH and the COMBINE Study

Because no single treatment approach is effective for everyone with alcohol dependence, clinicians and researchers proposed that assigning patients to treatment based on specific needs and characteristics would improve outcomes. NIAAA initiated Project MATCH in 1989 to test this theory. Patients—who were characterized according to factors such as severity of alcohol involvement, cognitive impairment, psychiatric severity, gender, motivational readiness to change, and social support for drinking versus abstinence—were randomly assigned to 12-step facilitation, cognitive–behavioral therapy, or motivational enhancement therapy. Patients were followed at 3-month intervals for 1 year after completion of the 12-week treatment period and were evaluated for changes in drinking patterns, functional status/quality of life, and treatment services utilization. The study found that patients with low psychiatric severity were best suited to 12-step facilitation therapy. These patients had more abstinent days than those treated with cognitive–behavioral therapy. Overall, Project MATCH participants showed significant improvement in percentage of abstinent days and decreased number of drinks per drinking days, with few significant outcome differences among the three treatment groups (Project MATCH Research Group 1997).

Following Project MATCH, the next step for evaluating treatment options was a large-scale study of medications for alcohol dependence. Combining Medications and Behavioral Interventions for Alcoholism, or the COMBINE Study, evaluated the efficacy of naltrexone and acamprosate, both alone and in combination, with medical management (i.e., patients had brief sessions with a health care professional) with and without behavioral therapy. The behavioral treatment integrated aspects of cognitive–behavioral therapy, motivational interviewing, and 12-step facilitation. Patients who received naltrexone, behavioral therapy, or both demonstrated the best drinking outcomes after 16 weeks of treatment. Acamprosate showed no evidence of efficacy, with or without behavioral therapy (Anton et al. 2006).

In addition to naltrexone (and an injectable, long-acting form of naltrexone) and acamprosate, disulfiram (Antabuse^®^) also is approved to treat alcohol dependence. Naltrexone helps to reduce the craving for alcohol after someone has stopped drinking. Acamprosate is thought to work by reducing symptoms that follow lengthy abstinence, such as anxiety and insomnia. Disulfiram discourages drinking by making the patient feel sick after drinking alcohol. Other types of drugs are available to help manage symptoms of withdrawal.

As shown in COMBINE, no single medication or treatment strategy is effective in every case or in every person. As research exploring the neuroscience of alcoholism continues to pave the way for new medications, studies also have sought to better understand why some behavioral interventions are more effective than others. The articles to follow in this special issue examine a broad range of topics relevant to developing and applying new treatment tools and methods.

## Medications Management

With the high prevalence of AUDs in the United States and low rates of treatment seeking, the value of identifying and treating alcohol problems in primary care settings is well known. As Stephanie O’Malley, Ph.D., and Patrick G. O’Connor, M.D., M.P.H., report in their article to follow (pp. 300–312), it is important to have effective approaches available to assist patients identified in primary care. Multiple studies have supported the efficacy of brief-intervention counseling in primary care settings. Research also supports the use of medications in primary care and suggests that, with counseling, this approach to treating alcohol problems is cost-effective and facilitates patients receiving continuing care. In addition to the four medications currently approved for treating alcohol dependence, efforts are underway to identify new medications that may be more effective. Other medications with some clinical evidence of efficacy include topiramate (an antiseizure medication); selective serotonin reuptake inhibitors (approved for depression); ondansetron (a serotonin receptor antagonist approved for nausea); baclofen (a γ-aminobutyric acid-b receptor agonist used for muscle spasticity), and atypical neuroleptics such as aripiprozole and quetiapine. Nicotinic compounds, including agonists, partial agonists, and antagonists, currently are under investigation. In addition, researchers are evaluating the therapeutic potential of corticotrophin-releasing factor antagonists and neurokinin 1 antagonists, which may address the relationship between stress and alcohol consumption.

## Behavioral Therapy

All behavioral approaches to the treatment of AUDs combine general behavioral principles (e.g., reinforcement and punishment) with therapeutic techniques designed to facilitate healthy behavior change. Coping skills training, cognitive–behavioral treatment, brief interventions, and relapse prevention also introduce concepts from cognitive therapy and social-learning theory. For example, the cognitive concept of self-efficacy, or belief in one’s ability to abstain from alcohol, plays a prominent role in both cognitive–behavioral treatments and relapse prevention. Likewise, a person’s expectations regarding the effects of alcohol (i.e., expectancies) often are identified and challenged during the course of cognitive–behavioral interventions. Coping skills training and relapse prevention primarily focus on identifying high-risk situations for drinking and then building a repertoire of coping skills to help patients approach risky situations without using alcohol. Brief interventions also utilize many cognitive– behavioral tools; however, in these cases, treatment occurs over a short period of time (often an hour or less).

In their article on behavioral therapy, Katie Witkiewitz, Ph.D., and G. Alan Marlatt, Ph.D. (pp. 313–319) describe and report on the efficacy of interventions including contingency management; couples, marital, and family therapy; facilitated self-change; and brief intervention. All of these treatments can be delivered in individual sessions or group formats, and many of them have been adapted to be delivered in a variety of treatment settings, including residential, outpatient, computerized, medical, and workplace settings. New methods of delivery and successful adjuncts to existing behavioral treatments also have been developed, including computerized cognitive–behavioral treatments, Web-based, guided, self-change, and mindfulness-based approaches. Choosing the most appropriate treatment for a given patient remains a challenge. Although research in this area has previously focused on comparing the effectiveness of different therapies, it is important for future research to also consider how people change as well as the mechanisms of change at work during the course of behavioral treatments.

## The Use of Emerging Technologies in Alcohol Treatment

The delivery of alcohol treatment, whether that treatment is medication, behavioral therapy, or a combination of both, can be facilitated by the use of communication tools such as the telephone, e-mail, and the Internet. These tools also can be used to identify people with alcohol problems. In their article, John A. Cunningham, Ph.D., Kypros Kypri, Ph.D., and Jim McCambridge, Ph.D. (pp. 320–326), describe the growing use of emerging technologies or electronic tools used to provide services to help problem drinkers. Among the applications being used are Internet- and computer program–based screening instruments (e.g., www.AlcoholScreening.org), online social support groups, Internet-based interventions, telephone contact, e-mail, and text messaging.

Emerging technologies can be used in primary care, the emergency department, prenatal care settings, college settings, and traditional addiction-treatment settings. Research is needed to demonstrate efficacy, to explore how to use these tools most effectively, and how to integrate them into traditional treatment modalities.

In a sidebar to the topic of emerging technologies, David Gustafson, Ph.D., and colleagues (pp. 327–337) describes a cell phone–based support system to be given to patients as they leave residential treatment. The technology, called A-CHESS (Addiction Comprehensive Health Enhancement Support System), is designed to provide coping competence, social support, and autonomous motivation. A-CHESS contains a proactive computer-based relapse prevention system, data transfer from A-CHESS to a care manager’s computer, vehicles for the patient to maintain contact with his/her care manager, audio/visual delivery of content to provide access to those with reading difficulties, and anywhere/anytime access through a smartphone. The researchers hypothesize that A-CHESS will reduce days of risky drinking by reducing negative affect, which will be mediated by social support, autonomous motivation, and improved coping strategies.

## Integrating Care for People With Co-Occurring Alcohol and Other Drug, Medical, and Mental Health Conditions

Treatment support and management is especially important for people with AUDs and co-occurring disorders (CODs) such as mental health and medical problems. Stacy Sterling, M.P.H., M.S.W.; Felicia Chi, M.P.H.; and Agatha Hinman (pp. 338–349), state in their article that care for patients’ AUDs, mental health, and medical problems primarily is provided in separate treatment systems and integrated care addressing all of a patient’s CODs in a coordinated fashion is the exception in most settings. A variety of barriers impede further integration of care for patients with CODs. These include differences in education and training of providers in the different fields, organizational factors, existing financing mechanisms, and the stigma still often associated with AUDs and CODs. Many programs are recognizing the disadvantages of separate treatment systems and are attempting to increase integrated approaches. Although few studies have been done in this field, findings suggest that patients receiving integrated treatment may have improved outcomes.

## The Role of Mutual-Help Groups in Extending the Framework of Treatment

Despite the advances in pharmacological and behavioral treatment reported throughout this issue, peer-run mutual-help groups (MHGs) such as AA continue to play an important role in helping millions of Americans achieve recovery. Indeed, MHGs are the most commonly sought source of help for alcohol and drug use problems in the United States. In their article, John F. Kelly, Ph.D., and Julie D. Yeterian (pp. 350– 355) describe the nature and prevalence of MHGs, particularly AA, and review evidence for their effectiveness, cost-effectiveness, and for the mechanisms through which they may exert their effects. The article also provides details about how health care professionals, including primary care providers, can facilitate their alcohol-dependent patients’ participation in such groups and reviews the evidence for the benefits of doing so. In contrast to professional treatments, people typically have access to MHGs at times when they are at higher risk of relapse, such as evenings and weekends, and many MHGs encourage members to contact each other by phone between meetings whenever help is needed. Consequently, these organizations provide an adaptive community-based system that is highly responsive to changes in relapse risk.

## Treating Alcoholism As a Chronic Disease: Approaches to Long-Term Continuing Care

MHGs can be especially valuable for patients with chronic, recurring AUDs involving multiple cycles of treatment, abstinence, and relapse. James R. McKay, Ph.D., and Susanne Hiller-Sturmhöfel, Ph.D. (pp. 356–370), describe in their article how efforts to disrupt this cycle can include strategies to reduce the risk of relapse, including initial intensive inpatient or outpatient care based on 12-step principles, followed by continuing care involving MHGs, 12-step group counseling, or individual therapy. Although these programs can be effective, many patients drop out of initial treatment or do not complete continuing care. Thus, researchers and clinicians have begun to develop alternative approaches to enhance treatment retention in both initial and continuing care. One focus of these efforts has been the design of extended treatment models. These approaches increasingly blur the distinction between initial and continuing care and aim to prolong treatment participation by providing a continuum of care. Other researchers have focused on developing alternative treatment strategies (e.g., telephone-based interventions) that go beyond traditional settings.

## The Recovery Spectrum: From Self-Change to Seeking Treatment

As reported above, a large percentage of people with AUDs go untreated in the United States. Jalie A. Tucker, Ph.D., M.P.H., and Cathy A. Simpson, Ph.D. (pp. 371–379) explain in their article that most people with alcohol problems recognize their situation long before they seek treatment, implying that interventions could be provided earlier. Closing the gap between treatment need and service utilization therefore is a public health priority that depends on understanding relationships between help-seeking and recovery patterns and processes at both the population and individual levels. The authors suggest that a spectrum of services—including screening and brief intervention, guided self-change programs, and e-health options—is needed to match the needs and preferences of the under-treated population.

In a sidebar to this article, Robert J. Meyers, Ph.D., Hendrik G. Roozen, Ph.D., and Jane Ellen Smith, Ph.D. (pp. 380–388) describe the community reinforcement approach (CRA), which helps people rearrange their lifestyles so that healthy, drug-free living becomes rewarding and thereby competes with substance use. This approach also encourages people to become progressively involved in alternative non–substance-related social activities and to focus on the enjoyment of work and family activities. A variation of CRA, the community reinforcement and family-training approach, works through friends and family members promoting treatment entry for treatment-resistant individuals.

## Health Services and Financing of Treatment

Recognizing the need for treatment and finding an appropriate treatment setting and provider are important steps in the recovery process. Another important factor influencing treatment access is cost and how services are financed and paid for. Since the 1960s, changes in these factors have driven changes in the delivery of treatment. Recent developments, including the passage of Federal parity legislation and health care reform, as well as increasing use of performance contracting, promise to bring additional changes. In their article, Maureen T. Stewart, Ph.D., and Constance M. Horgan, Sc.D. (pp. 389– 394) outline the current state of the substance abuse treatment system and highlight implications of these impending changes for access to and quality of treatment services. With the rise of managed care, private insurance coverage has been declining as a share of total treatment expenditures since 1986. People without insurance coverage or with limited insurance coverage for substance abuse treatment can pay out of pocket for services or through publicly funded addiction treatment programs. Performance-based contracts have been implemented to try to improve program accountability and provide incentives for high-quality care by tracking activities that are thought to facilitate positive patient outcomes.

The Federal parity law was designed to remove barriers to utilization, remove financial burdens on patients, and reduce stigma around addictive and mental disorders by requiring group health plans that offer mental health/addiction services to cover these services in a comparable manner to medical/surgical services. This is likely to result in changes to the management of treatment services under private and public insurance, as insurers will have to apply similar processes to medical and behavioral health care. National health care reform will increase insurance access by expanding Medicaid eligibility and mandating individual insurance coverage.

## Conclusion

Treatment for AUDs has made significant advances in the last 20 years. Researchers are working on numerous and novel approaches to improving the effectiveness, accessibility, quality, and cost-effectiveness of treatment. Practitioners now have at their disposal a full menu of evidence-based options to treat AUDs. In addition, recent work on the organization and delivery of alcohol services will play an increasingly important role as health care reform unfolds. This domain of alcohol research will not only reveal new medications and behavioral therapies, but will also lay the foundation for the development of exciting and potentially radical new approaches to a longstanding public health problem.
